# The Impact of Flow-Mediated Vasodilatation on Mechanism and Prognosis in Patients with Acute Coronary Syndrome: A FMD and OCT Study

**DOI:** 10.31083/j.rcm2504123

**Published:** 2024-03-28

**Authors:** Bin Zhu, Qiuwen Wu, Kunlei Yan, Gang Liu, Haibo Jia, Sining Hu, Fan Wang, Wei Meng, Ming Zeng, Xi Chen, Bo Yu, Shuo Zhang

**Affiliations:** ^1^Department of Cardiology, The 2nd Affiliated Hospital of Harbin Medical University, 150086 Harbin, Heilongjiang, China; ^2^The Key Laboratory of Medical Ischemia, Chinese Ministry of Education, 150086 Harbin, Heilongjiang, China

**Keywords:** acute coronary syndrome, flow-mediated vasodilatation, optical coherence tomography, plaque rupture, outcomes

## Abstract

**Background:**

Endothelial dysfunction, characterized by impaired flow-mediated vasodilation (FMD), is associated with atherosclerosis. However, the relationship between FMD, plaque morphology, and clinical outcomes in patients with acute coronary syndrome (ACS) remains underexplored. This study aims to investigate the influence of FMD on the morphology of culprit plaques and subsequent clinical outcomes in patients with ACS.

**Methods:**

This study enrolled 426 of 2482 patients who presented with ACS and subsequently underwent both preintervention FMD and optical coherence tomography (OCT) between May 2020 and July 2022. Impaired FMD was defined as an FMD% less than 7.0%. Major adverse cardiac events (MACEs) included cardiac death, nonfatal myocardial infarction, revascularization, or rehospitalization for angina.

**Results:**

Within a one-year follow-up, 34 (8.0%) patients experienced MACEs. The median FMD% was 4.0 (interquartile range 2.6–7.0). Among the patients, 225 (52.8%) were diagnosed with plaque rupture (PR), 161 (37.8%) with plaque erosion (PE), and 25 (5.9%) with calcified nodules (CN). Impaired FMD was found to be associated with plaque rupture (odds ratio [OR] = 4.22, 95% confidence interval [CI]: 2.07–6.72, *p* = 0.012) after adjusting for potential confounding factors. Furthermore, impaired FMD was linked to an increased incidence of MACEs (hazard ratio [HR] = 3.12, 95% CI: 1.27–6.58, *p* = 0.039).

**Conclusions:**

Impaired FMD was observed in three quarters of ACS patients and can serve as a noninvasive predictor of plaque rupture and risk for future adverse cardiac outcomes.

## 1. Introduction

While treatment options for acute coronary syndrome (ACS) have improved over the last few decades, rates of morbidity and mortality remain high, creating substantial health and economic challenges [1, 2]. Thrombotic occlusion, due to plaque rupture (PR) and plaque erosion (PE), is responsible for up to 90% of ACS cases, often leading to myocardial infarction or injury [3, 4, 5]. While early revascularization by stenting is the standard recommendation for patients with ACS, recent studies suggest that conservative treatment may be a viable alternative to stent implantation for patients with PE [6, 7]. Consequently, there is a need for reliable noninvasive predictors of PR and PE to tailor individual treatment approaches and reduce the likelihood of adverse events.

Flow-mediated vasodilation (FMD) is a noninvasive ultrasound technique for quantifying endothelial function [8]. A lower FMD rate is associated with a worse prognosis, and more severe lesions [9, 10, 11]. There is growing evidence suggesting that endothelial dysfunction contributes to atherogenesis and thrombosis, potentially predisposing individuals to PR [12, 13]. However, there is a notable lack of evidence linking endothelial function with the onset of PR and PE.

Optical coherence tomography (OCT) is a high-resolution intracoronary imaging technique that accurately identifies the underlying ACS pathology ACS. However, the relationship between plaque morphologies and endothelial dysfunction remains largely unexplored. Therefore, this study aims to identify the pathological mechanisms and plaque characteristics of ACS patients with impaired FMD compared with those with normal FMD.

## 2. Methods

### 2.1 Study Population

Between May 2020 and July 2022, a total of 426 patients who presented with acute coronary syndrome (ACS) and underwent OCT and were subsequently examined with FMD. These patients were recruited from the Second Affiliated Hospital of Harbin Medical University in Harbin, China. STEMI, NSTEMI, and unstable angina were all identified as ACS. The criteria for the diagnosis of ACS have been described previously [5, 14]. The patients provided written informed consent, and the present study was approved by the Ethics Committee of the Second Hospital of Harbin Medical University (Harbin, China).

### 2.2 Measurement of FMD

B-mode ultrasound images (UNEX EF; Unex Co., Ltd., Nagoya, Japan) were used to measure vasodilator responses in brachial arteries, as described in previous studies [8, 9]. Patients were required to fast for at least 6 h prior to vascular scans. The measurement of FMD was required before coronary intervention, unless it conflicted the guideline-recommended therapy strategy. In these cases, FMD measurements were permitted within 2 weeks of hospitalization. The standard FMD measurement algorithm was based on expert consensus guideline for reducing variations in the process of FMD measurement [13]. The ultrasound probe was placed between 1 and 5 cm above the brachial artery to obtain optimal FMD images for all patients. Vessel diameter and blood flow responses to reactive hyperemia and nitroglycerin were expressed as percentage increases in from their respective baseline values. Impaired FMD was defined as <7.0% (calculated as the mean minus one standard deviation of FMD).

### 2.3 Coronary Angiography Analysis

The Cardiovascular Angiography Analysis System (CAAS), version 5.10 (Pie Medical Imaging B.V., Maastricht, Netherlands) was used to perform quantitative coronary angiography (QCA) analysis. The QCA parameters, including reference vessel diameter, minimal lumen diameter, diameter stenosis, and lesion length, were measured as described in a previous study [15]. The culprit artery was determined based on the severity of the angiographic atherosclerosis, ECG changes, and OCT findings.

### 2.4 OCT Acquisition and Analysis

OCT imaging was performed using the commercially available C7-XR/ILUMIEN OCT system (Abbott Vascular, Santa Clara, CA, USA). The decision to perform OCT imaging was based on the operator’s discretion without prespecified angiographic or FMD demands. OCT imaging was routinely performed in most ACS patients except those with renal dysfunction, or unstable hemodynamics. OCT analyses were independently performed by two investigators (B.Z. and K.Y.) who were blinded to the clinical, angiographic, laboratory, and FMD data using an offline review workstation (Abbott Vascular). Any discordance was resolved by consensus with a third reviewer (W.M.). Quantitative and qualitative analyses of all lesions were performed as previously described [15]. To identify the culprit lesions, angiography, electrogram changes and/or left ventricular wall motion abnormalities were collectively evaluated. Quantitative analysis was performed using 1-mm intervals of cross-sectional OCT images. PE were identified by the presence of attached thrombi overlying an intact and visible plaque, an irregular luminal surface without thrombi, superficial lipid, or calcification immediately accompanied by attenuation of the underlying plaque by a thrombus. PR was characterized by a discontinuous fibrous cap with an intraplaque cavity [4, 15, 16].

### 2.5 Clinical Outcomes

All patients were followed for 1, 3, 6 and 12 months and subsequently annually by phone or hospital visits. Major adverse cardiovascular events (MACEs) were defined as a composite of cardiac death, nonfatal myocardial infarction, clinical-driven revascularization, and rehospitalization for unstable or progressive angina. All events were adjudicated by the independent Clinical Events Committee (CEC) of the Second Affiliated Hospital of Harbin Medical University.

### 2.6 Statistical Analysis

Statistical analysis was performed using the SPSS software (SPSS version 23.0, IBM, Armonk, New York, USA). Data distribution was assessed using the Kolmogorov–Smirnov test. Normally distributed continuous variables are presented as mean ± standard deviation and examined using Student’s *t*-test. Non-normally distributed continuous variables are presented as medians (interquartile ranges) and examined using the Mann–Whitney U test. Categorical data are presented as counts (proportions) and were compared using the chi-square test or Fisher’s exact test. The association of demographic and traditional risk factors, plaque characteristics, FMD, and culprit mechanisms (PE/PR) was analyzed using a multivariable logistic regression model with stepwise selection of the variable (*p* < 0.1 in the univariate analysis). The predictability of PR or thin cap fibroatheroma (TCFA) with FMD was determined by receiver operating characteristics curves analysis. Kaplan-Meier analysis was used to present time-to-event data and compared by log-rank test. The predictor of MACEs was identified by multivariable Cox regression model. A two-sided *p*-value < 0.05 was considered statistically significant.

## 3. Results

### 3.1 Demographics and Angiographic Findings

**Fig. 1.**
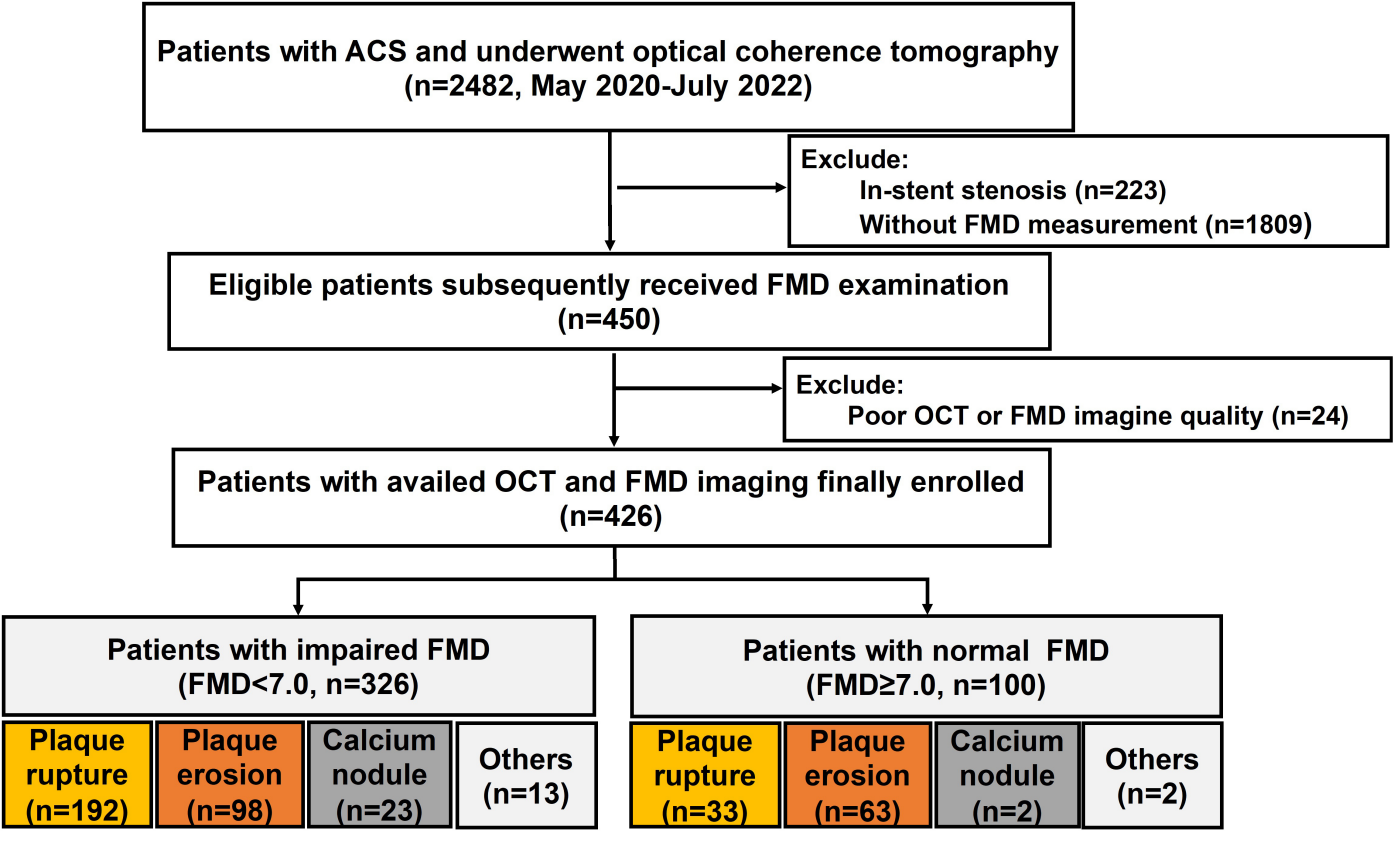
**Inclusion and exclusion criteria study flow-chart**. Between May 2020 and July 2022, 426 patients who underwent both FMD and OCT were included in the final analysis. Notably, over half of patients with impaired FMD (FMD <7.0%) exhibited plaque rupture. ACS, acute coronary syndrome; OCT, optical coherence tomography; FMD, flow-mediated vasodilation.

Patients undergoing both OCT and FMD testing (n = 426), were recruited between May 2020 to July 2022 and subsequently included in the final analysis. The detailed inclusion and exclusion criteria are shown in Fig. 1. Of these patients, 326 (76.5%) presented with impaired FMD, while 100 (23.5%) patients presented with normal FMD, as summarized in Table 1. The baseline clinical characteristics showed no significant demographic differences between the impaired and normal FMD groups, except for hypertension (69.6% vs. 53.0%, *p* = 0.002). Patients with impaired FMD exhibited non-significant trends towards both older age (60.0 years vs. 56.4 years, *p* = 0.053) and higher LDL-C levels (2.2 mmol/L vs. 2.0 mmol/L, respectively; *p* = 0.052) than those with normal FMD.

**Table 1.** **Baseline clinical characteristics**.

		All patients	Impaired FMD	Normal FMD	*p* value
		(n = 426)	(n = 326)	(n = 100)	
Age, yrs		59.1 ± 10.5	60.0 ± 10.5	56.4 ± 10.3	0.053
Male gender (%)		307 (72.1)	241 (73.9)	66 (66.0)	0.122
BMI, ${\mathrm{kg/m}}^{2}$		26.2 ± 3.3	26.2 ± 3.2	26.4 ± 3.6	0.233
Risk factor					
	Current Smoking (%)	310 (72.8)	235 (78.6)	75 (75.0)	0.567
	Diabetes mellitus (%)	142 (33.3)	109 (33.4)	33 (33.0)	0.936
	Hyperlipidemia (%)	159 (37.4)	126 (38.8)	33 (33.0)	0.297
	Hypertension (%)	280 (65.7)	227 (69.6)	53 (53.0)	0.002
	Chronic kidney disease (%)	7 (1.6)	5 (1.5)	2 (2.0)	0.667
Prior history					
	Prior MI (%)	31 (7.3)	25 (7.7)	6 (6.0)	1.000
	Prior PCI (%)	110 (25.8)	83 (25.5)	27 (27.0)	0.758
Clinical manifestation					
	STEMI	234 (54.9)	176 (54.0)	58 (58.0)	0.889
	NSTEMI	128 (30.0)	96 (29.3)	32 (32.0)	
	UAP	64 (15.0)	47 (14.4)	17 (17.0)	
LVEF, %		62.0 (61.0–64.0)	61.0 (60.0–64.0)	63.0 (62.0–64.0)	0.833
Brachial artery diameter, mm		4.2 ± 0.6	4.2 ± 0.6	4.1 ± 0.6	0.507
%FMD		4.0 (2.6–7.0)	3.2 (2.3–4.4)	7.6 (7.4–8.5)	<0.001
Laboratory variables					
	TC, mmol/L	4.5 (3.8–5.4)	4.6 (4.0–5.5)	4.4 (3.7–5.1)	0.217
	Triglyceride, mmol/L	1.4 (1.0–2.1)	1.3 (0.9–2.4)	1.5 (1.0–2.1)	0.832
	LDL–C, mmol/L	2.1 (1.7–2.9)	2.0 (1.5–2.6)	2.2 (1.7–3.0)	0.052
	HDL–C, mmol/L	1.0 (0.9–1.2)	1.0 (0.8–1.2)	1.0 (0.9–1.2)	0.408
	HbA1c (%)	6.0 (5.6–7.1)	5.9 (5.6–7.3)	6.1 (5.6–7.1)	0.696
	hs–CRP, mg/dL	4.1 (1.8–9.2)	4.2 (1.9–9.2)	4.0 (1.7–9.3)	0.678
	Peak TnI, ug/L	22.1 (2.7–82.3)	20.6 (2.3–71.8)	25.6 (3.4–91.2)	0.850

Values are n (%), mean ± SD or median (interquartile range). BMI, body mass index; MI, myocardial infarction; LVEF, left ventricular ejection fractions; STEMI, ST segment elevation myocardial infarction; NSTEMI, Non-ST-segment elevation myocardial infarction; UAP, unstable angina pectoris; FMD, flow-mediated vasodilatation; TC, total cholesterol; LDL-C, low-density lipoprotein cholesterol; HDL-C, high-density lipoprotein cholesterol; hs-CRP, high-sensitive C-reactive protein. TnI, troponin I; PCI, percutaneous coronary intervention.

The majority of affected vessels (53.1%) were found in the left anterior descending artery. The culprit artery locations evenly distributed between the left anterior descending (53.4% vs. 52.0%, respectively), left circumflex (20.9% vs. 23.0%, respectively), and right coronary arteries (25.8% vs. 25.0%, respectively). There were no significant differences in quantitative coronary analysis in terms of reference vessel diameter, minimal lumen diameter, diameter stenosis, and lesion length (Table 2).

**Table 2.** **Angiographic findings**.

		All patients	Impaired FMD	Normal FMD	*p* value
		(n = 426)	(n = 326)	(n = 100)	
Culprit location					0.901
	LAD (%)	226 (53.1)	174 (53.4)	52 (52.0)	
	LCX (%)	91 (21.4)	68 (20.9)	23 (23.0)	
	RCA (%)	109 (25.6)	84 (25.8)	25 (25.0)	
Quantitative coronary analysis					
	RVD, mm	2.8 ± 0.7	2.7 ± 0.7	2.9 ± 0.8	0.318
	MLD, mm	1.0 ± 0.4	1.0 ± 0.4	1.1 ± 0.5	0.170
	DS, %	63.7 ± 14.6	61.5 ± 17.7	64.4 ± 13.5	0.265
	Lesion length	10.5 (7.4–14.9)	10.6 (7.8–15.1)	10.4 (7.0–14.5)	0.510

Values are n (%), mean ± SD or median (interquartile range). FMD, flow-mediated vasodilatation; LAD, left anterior descending; LCX, circumflex; RCA, right coronary artery; RVD, reference vessel diameter; MLD, minimal lumen diameter; DS, diameter stenosis.

### 3.2 Distribution of Different Levels of FMD

**Fig. 2.**
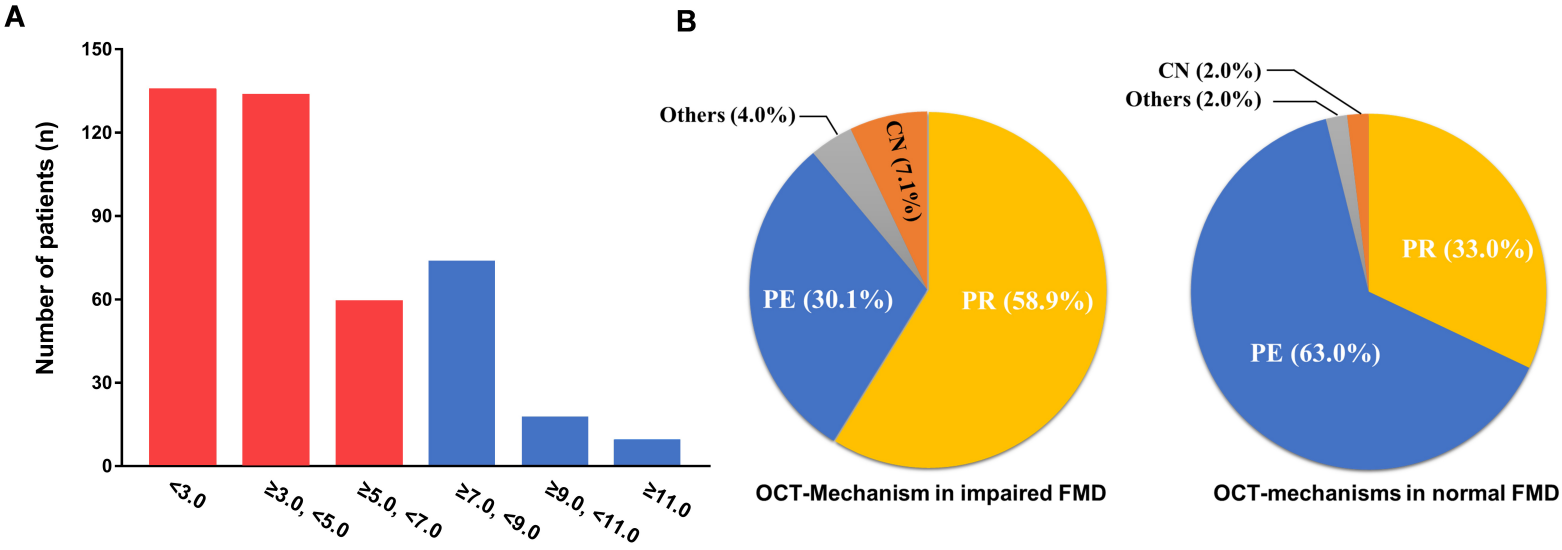
**Distribution of FMD spectra and their associated OCT-mechanism**. (A) Bar graph depicting the distribution of impaired FMD and non-impaired FMD in patients with ACS. Red indicates impaired FMD (<7.0%) and blue indicates non-impaired FMD (≥7.0%). (B) The left sector chart indicates the distribution of OCT mechanisms in patients with impaired FMD. The right-sector chart shows the distribution of OCT mechanisms in patients with normal FMD. ACS, acute coronary syndrome; OCT, optical coherence tomography; FMD, flow-mediated vasodilation; CN, calcified nodules; PR, plaque rupture; PE, plaque erosion.

The baseline brachial artery diameter was 4.2 ± 0.6 mm, with an average FMD of 4.0% (interquartile: 2.6–7.0%). Analysis of FMD indicated that 76.5% of the patients exhibited impaired FMD, defined as FMD <7.0%. Conversely, normal FMD (FMD ≥7.0%) was observed in 23.5% of patients. The distribution of the different spectra of the FMD is presented in Fig. 2.

### 3.3 Culprit Lesion’s Mechanisms

**Fig. 3.**
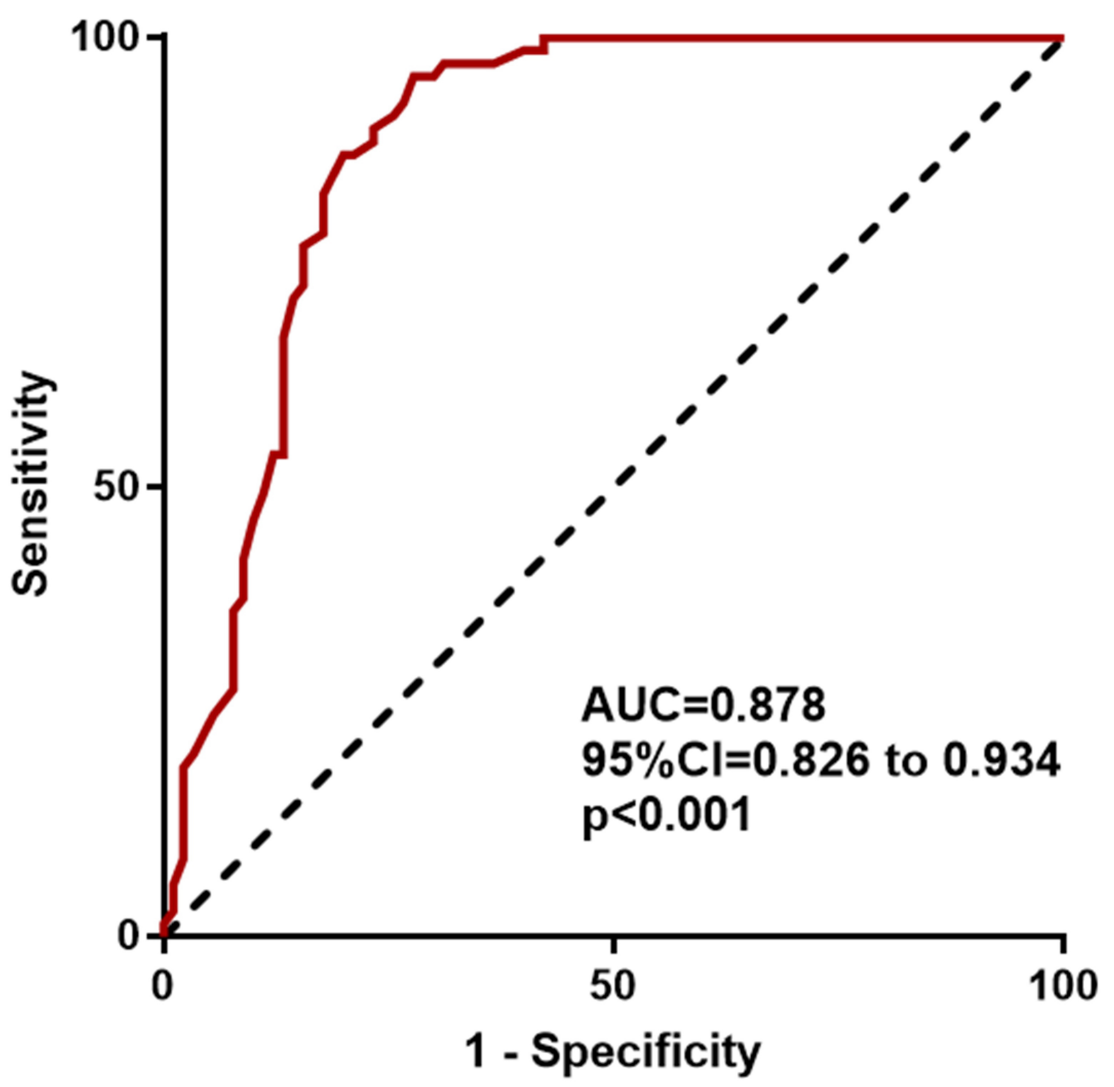
**Receiver operating characteristics curve analysis**. Receiver operating characteristics curve analysis to predict PR from FMD. The area under curve = 0.878, 95% confidential interval = 0.826–0.934, *p* < 0.001. PR, plaque rupture; FMD, flow-mediated vasodilation.

According to the established OCT criteria, within the study population, 225 (52.8%) experienced a PR, 161 (37.8%) experienced a PE, 25 (5.9%) experienced a calcified nodule, and 15 (3.5%) experienced other complications including 3 (0.7%) spasm, 6 (1.4%) SCAD and 6 (1.4%) tight stenosis. The details of the other mechanisms are presented in **Supplementary Table 1**. Patients with impaired FMD presented with PRs more frequently than those with normal FMD (58.9% vs. 33.0%, respectively), but this group also presented fewer incidences of PE (30.1% vs. 63.0%, respectively) (Fig. 2 and Table 3). Multivariable analysis revealed that patients with impaired FMD had a 4.2-fold higher risk of PR (odds ratio 4.22, 95% CI: 2.07–6.72; *p* = 0.012) than those with normal FMD, after adjusting for potential confounders (Table 4). The receiver operates characteristics (ROC) analysis demonstrated that impaired FMD could accurately predict PR (area under curve [AUC] = 0.878, 95% CI: 0.826–0.934, *p* < 0.001) (Fig. 3). Additionally, patients with impaired FMD were more likely to present with red thrombi compared to those with normal FMD (61.0% vs. 47.0%, respectively; *p* = 0.013). Representative cases illustrating the measurement of impaired FMD with OCT-based PR and normal FMD with OCT-based PE are presented in **Supplementary Figs. 1,2**.

**Table 3.** **Optical coherence tomography findings**.

		All patients	Impaired FMD	Normal FMD	*p* value
		(n = 426)	(n = 326)	(n = 100)	
Culprit mechanisms					<0.001
	Plaque rupture	225 (52.8)	192 (58.9)	33 (33.0)	
	Plaque erosion	161 (37.8)	98 (30.1)	63 (63.0)	
	Calcium nodule	25 (5.9)	23 (7.1)	2 (2.0)	
	Others	15 (3.5)	13 (4.0)	2 (2.0)	
Plaque features					
	Lipid plaque	225 (52.8)	180 (55.2)	45 (45.0)	0.073
	TCFA	105 (24.6)	92 (28.2)	13 (13.0)	0.002
	Lipid-rich plaque	138 (32.4)	119 (36.5)	19 (19.0)	0.001
	Cholesterol crystals	115 (27.0)	97 (29.8)	18 (18.0)	0.024
	Microchannel	73 (17.1)	58 (17.8)	15 (15.0)	0.517
	Calcification	76 (17.8)	57 (17.5)	19 (19.0)	0.729
	Macrophage	154 (36.2)	112 (34.4)	42 (42.0)	0.164
Thrombus					0.013
	Red thrombus	246 (57.7)	199 (61.0)	47 (47.0)	
	White thrombus	180 (42.3)	127 (39.0)	53 (53.0)	

Values are n (%). TCFA, thin cap fibroatheroma; FMD, flow-mediated vasodilatation.

**Table 4.** **Logistic regression analysis of impaired FMD for plaque rupture**.

Model	Odds Ratio (95% CI)	*p* value
Unadjusted	3.32 (1.15–5.69)	0.003
Model 1	3.28 (1.13–5.82)	0.017
Model 2	3.38 (1.16–5.93)	0.035
Model 3	4.22 (2.07–6.72)	0.012

Odds ratio shown were for Impaired FMD. Model 1 adjusted for age and sex; Model 2 adjusted for all factor in mode 1 plus smoking, diabetes; Model 3 adjusted for all factor in Model 2 plus TCFA, LRP, MLA <3.5 ${\mathrm{mm}}^{2}$ and cholesterol crystal. MLA, minimal lumen area; TCFA, thin-cap fibroatheroma; LRP, lipid-rich plaque; FMD, flow-mediated vasodilatation.

### 3.4 Plaque Vulnerability

**Fig. 4.**
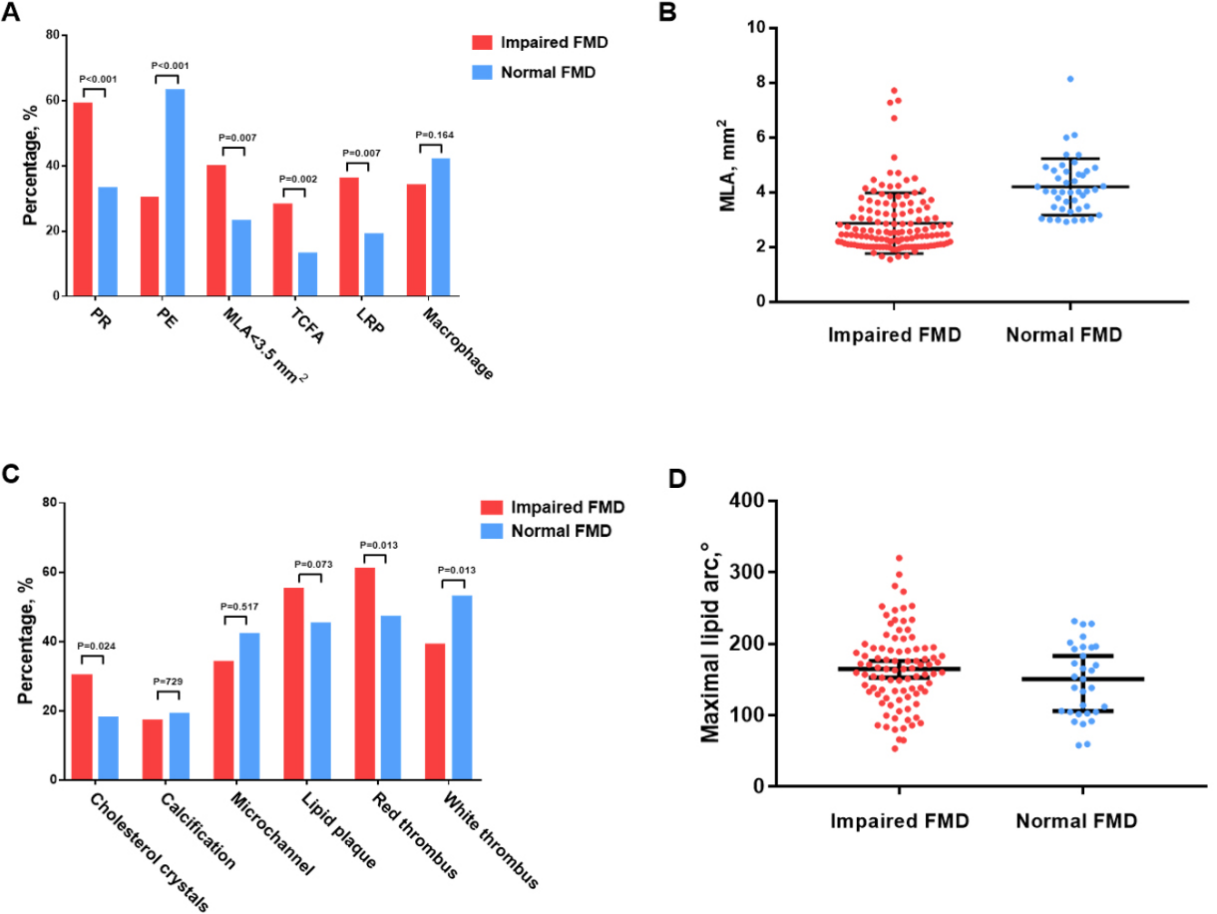
**Illustration of OCT findings, stratified by normal FMD versus impaired FMD**. OCT findings comparing impaired and normal FMD are presented: (A) culprit mechanisms and plaque characteristics, including PE, PR, MLA <3.5 ${\mathrm{mm}}^{2}$, TCFA, LRP, and macrophage; (B) MLA stratified by normal and impaired FMD; (C) other plaque characteristics, including cholesterol crystal, calcification, microchannel, lipid plaque, and red/white thrombus; (D) maximal lipid arc stratified by normal and impaired FMD. PR, plaque rupture; PE, plaque erosion; MLA, minimal lumen area; TCFA, thin-cap fibroatheroma; LRP, lipid-rich plaque; FMD, flow-mediated vasodilatation; OCT, optical coherence tomography.

Plaques in patients with impaired FMD were found to be more vulnerable, as defined by the presence of a fibro cap thickness (FCT) <65 µm (28.2% vs. 13.0%, respectively; *p* = 0.002) and minimal lumen area (MLA) <3.5 ${\mathrm{mm}}^{2}$ (46.0% vs. 23.0%, respectively; *p* = 0.007), when compared to patients with normal FMD (Table 3 and Fig. 4). Additionally, the presence of lipid-rich plaques was significantly higher in the impaired FMD group compared to the normal FMD group (36.5% vs. 19.0%, respectively; *p = *0.001). Notably, the impaired FMD score was moderately predictive of thin cap fibroatheroma (TCFA, AUC = 0.766, 95% CI: 0.691–0.840, *p* < 0.001) (**Supplementary Fig. 3**). No differences were observed in other plaque features, including cholesterol crystals, microchannels, calcification, and macrophages, and the proportion of lipid plaques were similar between the two groups (55.2% vs. 45.0%, respectively; *p* = 0.073).

### 3.5 Clinical Outcomes

**Fig. 5.**
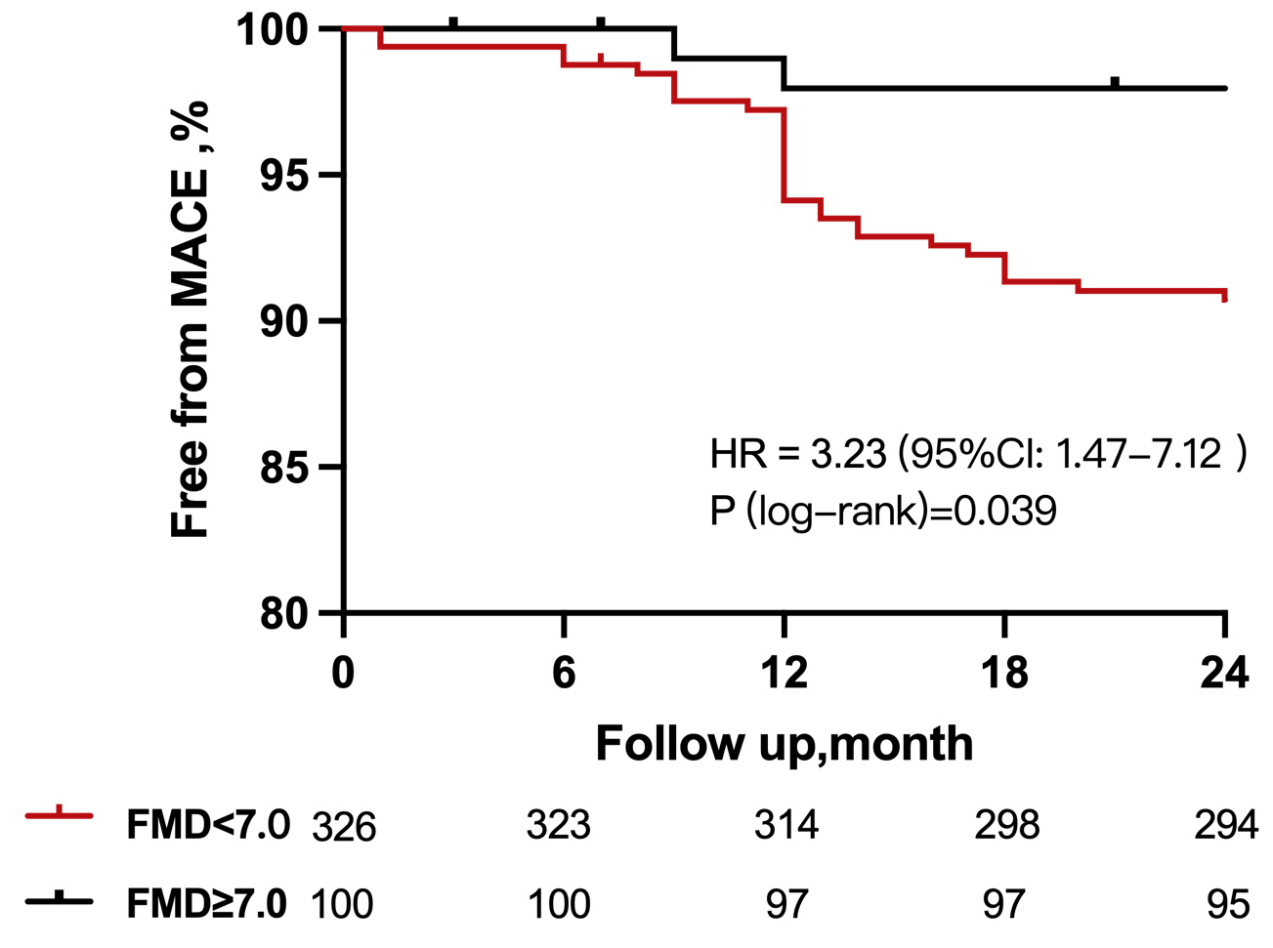
**Kaplan-Meier curves comparing MACE incidence based on FMD status**. There was a significant difference in MACE between patients with impaired FMD and normal FMD. MACE incidents included cardiac death, nonfatal myocardial infarction, revascularization, and rehospitalization for angina. MACE, major adverse cardiac event; FMD, flow-mediated vasodilatation; HR, hazard ratio.

All patients completed their scheduled one-year follow-up. The composite endpoint outcomes and their components are detailed in Table 5. The Kaplan-Meier curve shows the cumulative incidence of major adverse cardiac events (MACE) over time for the patients with impaired and normal FMD (Fig. 5). Incidences of MACEs occurred in 9.5% of patients with impaired FMD and 3.0% of patients with normal FMD (hazard ratio [HR] = 3.23, 95% CI: 1.47–7.12, *p* = 0.039). A multivariable Cox regression model revealed that impaired FMD was an independent predictor of adverse events (HR = 3.12, 95% CI: 1.27–6.58, *p* = 0.039) after controlling for potential confounding factors.

**Table 5.** **Clinical outcomes at the 12-month follow-up**.

Variable	All patients	Impaired FMD	Normal FMD	*p* value
	(n = 426)	(n = 326)	(n = 100)	
MACEs, n (%)	34 (8.0)	31 (9.5)	3 (3.0)	0.039
Cardiac death, n (%)	8 (1.9)	7 (2.1)	1 (1.0)	
Re-MI, n (%)	3 (0.7)	3 (0.9)	0 (0)	
Revascularization, n (%)	10 (2.3)	9 (2.8)	1 (1.0)	
Rehospitalization for progressive angina, n (%)	13 (3.1)	12 (3.7)	1 (1.0)	

MACEs occurred within 1 years including cardiac death, re-MI, revascularization, and rehospitalization for progressive angina. MACEs, major adverse cardiac events; MI, myocardial infarction; FMD, flow-mediated vasodilatation.

## 4. Discussion

To the best of our knowledge, this is the first observational study to compare the pathological mechanisms in culprit arteries of ACS patients with normal versus impaired FMD. The main findings are as follows. (i) Patients with impaired FMD are more likely to present with PR, suggesting that FMD may serve as a biomarker for differentiating between PR and PE. (ii) Impaired FMD was associated with increased culprit plaque vulnerability and unfavorable clinical outcomes.

### 4.1 Mechanism of FMD and Distribution in ACS

Microcirculatory dysfunction is linked to the development and progression of atherosclerosis and thrombosis. Diminished FMD may indicate systemic atherosclerotic risk, which consequently predicts adverse cardiovascular events. Endothelial vasodilation is largely mediated by nitro-oxide (NO); impairment of NO availability leads to endothelial dysfunction [8]. The response of vascular smooth cells is vital for FMD. Overexpression of peroxisome proliferator–activated receptor γ coactivator 1-α (PGC-1α) enhances NO and hydrogen peroxide during FMD [17]. Additionally, prostacyclin and NO are the main mediators of FMD in younger and older patients, respectively [18]. Therefore, understanding the diverse mechanisms regulating FMD, including the roles of NO and PGC-1α, is crucial for identifying potential therapeutic targets to mitigate systemic atherosclerotic risk and improve cardiovascular outcomes.

In a previous study, the FMD percentage was 7.6 ± 2.5 in patients with ACS, results that are higher than the data we have presented [19]. No significant difference in FMD was observed between patients with ACS and those with stable CAD [19]. However, Kitta *et al*. [20] reported that the baseline FMD was 3.0 ± 1.5% in patients with coronary artery disease (CAD). In another study, the baseline percentage of FMD was 2.1 ± 1.2% in patients with non-ST-elevated ACS [21]. Three-quarters of patients presenting with acute coronary syndrome after PCI were diagnosed with endothelial dysfunction, defined as FMD <7.0% (Figs. 1,2). These findings highlight the variability in FMD measurements across different patient populations and underscore the need for more standardized approaches to assess endothelial function, particularly in the context of ACS and CAD.

### 4.2 Impairment of FMD and Culprit Mechanism

Pathological PR and PE are the primary causes of ACS, having been reported in approximately 75% and 25% of ACS cases, respectively, aligning with our results [6, 22]. Jia *et al*. [3] first established OCT as an *in vivo* diagnostic algorithm for PE. Due to its high resolution (10–15 µm), OCT currently provides the best diagnostic imaging for PE [5, 23, 24, 25, 26]. Endothelial dysfunction, assessed by OCT-quantified FMD, may precede the asymptomatic vasculature atherosclerosis, potentially predicting future MACE events [19]. However, there is limited evidence of advanced atherosclerosis and endothelial dysfunction in patients with ACS.

This study is the first to highlight the increased risk of PR in patients with impaired FMD. The pro-inflammatory effects of endothelial dysfunction may be a contributing factor to the higher incidence of PR in these patients [27, 28]. This is supported by a previous study showing that impaired FMD was associated with severe coronary stenosis [29]. This suggests that patients with impaired FMD are more likely to experience PR, as severe atherosclerosis is more frequent in patients with PR than PE [4].

### 4.3 Impairment of FMD and Plaque Vulnerability

Because FMD impairment of can serve as an independent predictor of future adverse cardiovascular events, FMD screening may be an ideal tool for clinicians to develop both long-term and short-term risk management strategies. Emerging evidence suggests that high-risk plaque characteristics, such as TCFA, lipid-rich plaque, MLA <3.5 ${\mathrm{mm}}^{2}$, and a large plaque burden, can elevate the risk of major adverse events [30, 31, 32]. In patients with ACS and impaired FMD, the vascular structure exhibited increased plaque vulnerability, more TCFAs, and smaller MLA compared with those with unimpaired FMD. This increased vulnerability at the site of the culprit lesion may have systemic effects on pan-vascular plaque stability [15]. Therefore, FMD impairment is associated with greater plaque vulnerability and may lead to poor clinical outcomes in these at-risk patients.

### 4.4 Limitation

This study does have several limitations. First, as a retrospective single-center study, it may contain potential confounding factors related to the limited patient population. Second, FMD measurements were not performed routinely for all patients with ACS at the study center. Although no significant differences were observed between patients who underwent FMD measurement and the overall patient group, the non-routine nature of FMD measurements could introduce bias. However, not requiring target patients to undergo this examination might have partially reduced selection bias. Third, the OCT findings in non-culprit plaques were not analyzed due to the non-routine conduction of multivessel OCT for all patients. Finally, the lack of a uniform standard FMD measurement algorithm may have led to variations in the measurement. However, the FMD measurement protocol in this present study was based on updated consensus guidelines [13], bolstering confidence in our results and their applicability to clinical practice and future clinical trials.

## 5. Conclusions

Impaired FMD has been shown to predict PR and vulnerable plaque morphology in the ACS patient population. These results also correlated with poorer clinical outcomes. This suggests that FMD can serve as a noninvasive biomarker for predicting plaque morphology and identifying patients at high risk of recurrent adverse events.

## Data Availability

The datasets used and/or analyzed during the current study are available from the corresponding author on reasonable request.

## Abbreviations

ACS, acute coronary syndrome; CAD, coronary artery disease; FMD, flow-mediated vasodilatation; HR, hazard ratio; LRP, lipid-rich plaque; MLA, minimal lumen area; NSTEMI, non-ST segment elevated myocardial infarction; OCT, optical coherence tomography; PCI, percutaneous coronary intervention; PE, plaque erosion; PR, plaque rupture; STEMI, ST segment elevated myocardial infarction; TCFA, thin-cap fibroatheroma.

## Supplementary Material

Supplementary material associated with this article can be found, in the online version, at https://doi.org/10.31083/j.rcm2504123.
