# Supplementary material for: The Impact of Flow-Mediated Vasodilatation on Mechanism and Prognosis in Patients with Acute Coronary Syndrome: A FMD and OCT Study
Source: Rev Cardiovasc Med. 2024 Mar 28;25(4):123. doi: 10.31083/j.rcm2504123 (PMC11263997; doi:10.31083/j.rcm2504123)
Supplement: Supplementary file 1 [file 2153-8174-25-4-123-s1.docx]

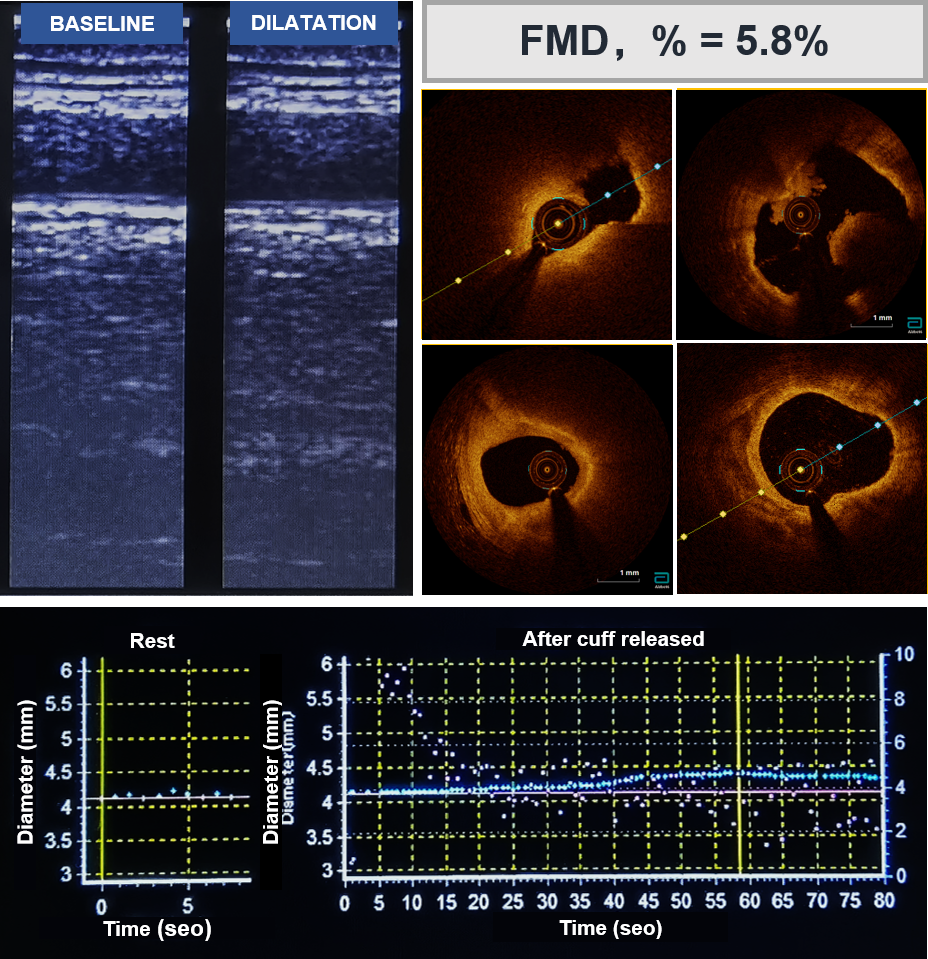


**Supplementary Fig. 1. Representative case for patient with impaired FMD and plaque rupture.**

**
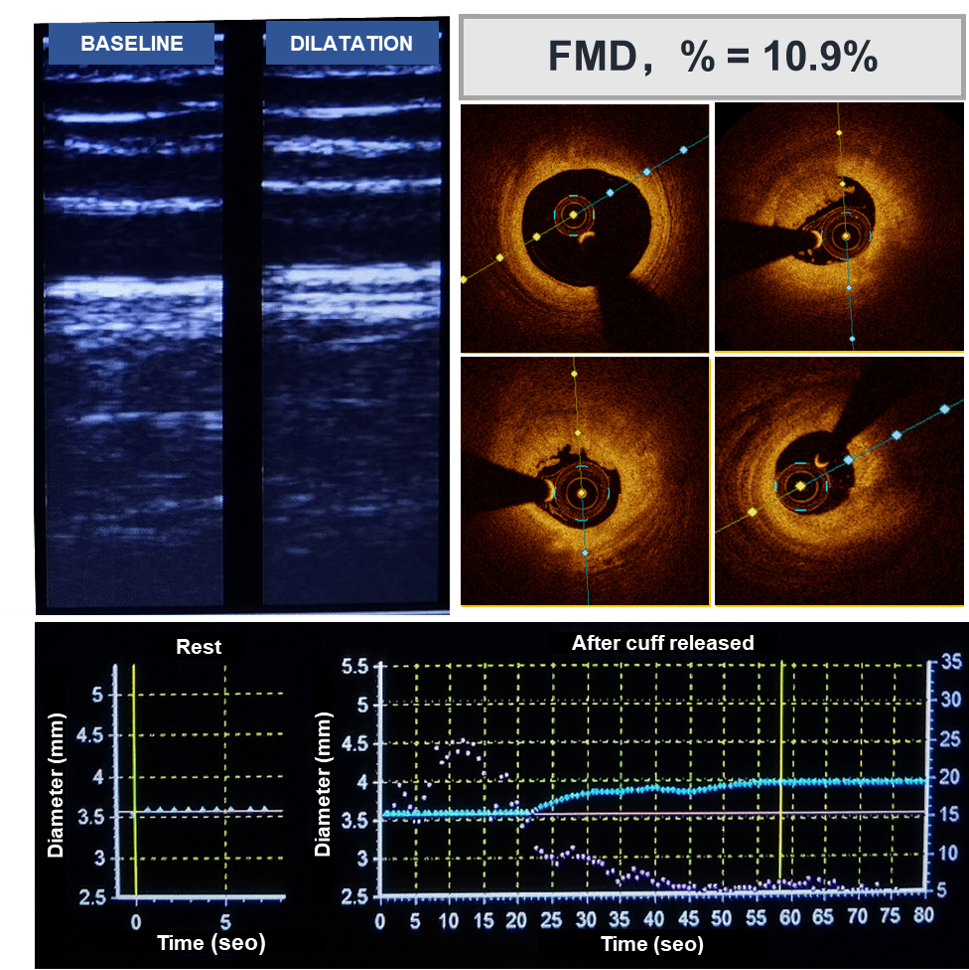
**

**Supplementary Fig. 2. Representative case for patient with normal FMD and plaque erosion.**

**
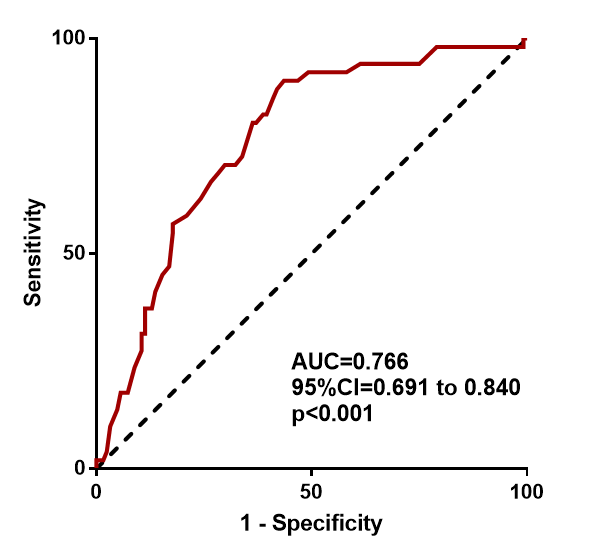
**

**Supplementary Fig. 3. Receiver operating characteristics curve analysis for thin cap fibroatheroma (TCFA).**

|  | All patients  (n=426) | Impaired FMD  (n=326) | Normal FMD  (n=100) |
| --- | --- | --- | --- |
| Other culprit mechanisms |  |  |  |
| Spasm | 3 (0.7) | 3 (0.9) | 0 (0) |
| SACD | 6 (1.4) | 5 (1.5) | 1 (1.0) |
| Tight stenosis | 6 (1.4) | 5 (1.5) | 1 (1.0) |
| Values are n (%).  SCAD = spontaneous coronary artery dissection; FMD = flow-mediated vasodilatation | | | |

**Supplemental Table 1. Distribution of Other Culprit Mechanisms.**
